# Ethical considerations of artificial intelligence in dementia research: a review of reviews

**DOI:** 10.1093/geront/gnag158

**Published:** 2026-07-23

**Authors:** Eunjung Ko, Hannah Cho

**Affiliations:** Rory Meyers College of Nursing, New York University, New York, New York, United States; School of Nursing, Stony Brook University, Stony Brook, New York, United States; School of Nursing, University of Pennsylvania, Philadelphia, Pennsylvania, United States; Mo-Im Kim Nursing Research Institute, College of Nursing, Yonsei University, Seoul, South Korea

**Keywords:** Dementia care, Artificial intelligence, Ethics, A review of reviews

## Abstract

**Background and Objectives:**

To examine the ethical considerations related to artificial intelligence technologies in dementia across diagnostic, management, and to synthesize these considerations using existing artificial intelligence and bioethical ethics principles.

**Research Design and Methods:**

The review of reviews synthesized evidence from published reviews addressing ethical considerations in published review-level evidence on ethical issues related to artificial intelligence for dementia and Alzheimer’s disease. Findings were extracted and narratively synthesized. Ethical issues were mapped to the four UNESCO value domains and four traditional bioethical principles to provide an interpretive framework.

**Results:**

Eighteen reviews were included. Ethical concerns were related to algorithmic bias, limited diverse data, privacy, data security, data governance, fairness, and psychosocioeconomic and environmental well-being, and caregiving contexts. Based on UNESCO’s four domains, six themes were identified: protection of human rights, technical trustworthiness, respect for human dignity, equity and access, governance, and environmental well-being. Ethical concerns prioritized the respect and protection of human rights and dignity, whereas environmental and ecosystem considerations were under focused. While many categories aligned with the traditional bioethical principles of autonomy, beneficence, non-maleficence, and justice, some concerns related to environmental well-being and data governance were difficult to classify within this framework.

**Discussion and Implications:**

Our findings highlight the importance of embedding ethical principles in individual-level and system-level design implications to support the ethical and sustainable use of artificial intelligence in dementia care. This review will help healthcare professionals understand and address the ethical considerations in various dementia care settings where artificial intelligence is introduced or evaluated.

## Background

In the context of dementia care, Artificial Intelligence (AI)-based approaches have increasingly been applied in healthcare to analyze complex datasets and support early detection, prediction, and monitoring of cognitive decline and dementia-related conditions (Angus et al., 2025; Graham et al., 2020; Habbal et al., 2025). These technologies include machine learning, deep learning, natural language processing, and large language models, which enable automated pattern recognition and data-driven decision-making across diverse health data sources. Recently, generative AI—capable of creating novel text, audio, and visual content—has begun to play a more active role in dementia care (Doshi & Hauser, 2024). It ranges from conversational agents that provide cognitive stimulation and companionship to systems that assist caregivers by synthesizing patient history and generating tailored care plans (Doshi & Hauser, 2024; Morris et al., 2025; Qiu et al., 2025). This shift in care approach has the potential to significantly enhance patients’ quality of life and alleviate the growing burden on healthcare systems and caregivers.

However, integrating AI into dementia care may introduce profound ethical challenges that must be rigorously addressed. As people living with dementia often face progressive declines in cognitive function and decision-making capacity, deploying AI interventions raises critical questions about informed consent, data privacy, and the risk of algorithmic system support rather than undermining their personal agency (Dino et al., 2025). Furthermore, a recent study highlights the concept of AI psychosis, illustrating how sustained engagement with conversational AI systems might trigger, amplify, or reshape altered perceptions of reality (Lawrence et al., 2024). Crucially, these ethical and practical considerations extend to caregivers. Many caregivers may face barriers related to digital health literacy; without inclusive design and skill-building, these innovative technologies risk excluding the very support networks they aim to assist, thereby exacerbating existing digital divides (Soares et al., 2024).

These concerns highlight the need for an ethical framework that can examine AI-related risks in dementia care. One widely adopted framework in the field of bioethics is the four principles developed by Beauchamp and Childress (1994): respect for autonomy, nonmaleficence, beneficence, and justice. Respect for autonomy ensures an individual’s right to self-rule, completely free from controlling interference by others. When it comes to treatment, however, we must balance nonmaleficence, the strict obligation to do no harm by intentionally abstaining from harmful action, with beneficence, which requires positive, deliberate action to benefit others. Finally, justice bridges these responsibilities by ensuring fairness, meaning that medical resources, benefits, and inherent risks are distributed equitably among all individuals without discrimination. These principles have been suggested as a useful foundation for evaluating ethical issues related to AI in healthcare (Gorelik et al., 2025). However, AI may cause additional ethical challenges that may not be fully captured by traditional bioethical principles alone. For example, Benzinger et al. (2023) used explicability alongside the traditional four principles based on Ursin et al. (2022)’s recommendation to examine ethical decision-making related to AI; still, there was limited attention to explicability, justice, and other concerns such as fairness and accountability.

Based on this need for a broader AI ethics framework in dementia care, this review adopts the foundational values outlined in the United Nations Educational, Scientific, and Cultural Organization (UNESCO) Recommendation on the Ethics of Artificial Intelligence as a guiding framework (UNESCO, 2022). Specifically, our analysis is based on four core values of the AI system life cycle. First, the ‘Respect, protection and promotion of human rights and fundamental freedoms and human dignity’ refers to individuals receiving AI-assisted care, who must never be objectified, subordinated to algorithmic outputs, or placed in situations that compromise their inherent dignity. Second, ‘Ensuring diversity and inclusiveness’ underscores the need to address disparities in technological infrastructure and digital literacy so that neither person living with dementia nor their caregivers are excluded from or disadvantaged by emerging interventions. Third, the value of ‘Living in peaceful, just, and interconnected societies’ highlights that AI systems should meaningfully support relational care and social connectedness, rather than fragment care networks or constrain autonomous decision-making. The last value, ‘Environment and ecosystem flourishing,’ refers to the long-term sustainability of technological innovation, which is not peripheral but a foundational value to everyone and benefits all living creatures. Guided by these values, this review of reviews synthesizes existing evidence on the use of AI in dementia care and examines how current literature aligns with, advances, or challenges these ethical values.

Despite the rapid expansion of research on AI in dementia care, the existing literature reviews on ethical considerations of AI vary substantially in scope, methodology, and conceptual focus. Many reviews concentrate on specific technological applications (e.g., conversational agents or predictive analytics) or short-term clinical outcomes, while providing limited examination of caregiver experiences, implementation strategies, and long-term ethical implications (Al-Nafjan et al., 2025; Rahsepar Meadi et al., 2025). Moreover, differences in outcome measures and inclusion criteria make it difficult to draw coherent conclusions across reviews. For instance, a past systematic review of the role of AI applications in dementia research highlighted gaps in how AI is studied and suggested future research directions should focus on consistent outcomes across technologies and use cases (Xie et al., 2020). Additionally, recent reviews of AI in healthcare (Benzinger et al., 2023; Gorelik et al., 2025) broadly explored overarching healthcare, not specifically focused on dementia care or research. Thus, there are still gaps in the literature that none of the literature reviews explored the reviews particularly AI ethical considerations in dementia research and care.

## Aim(s)

This review of reviews aims to: 1) examine ethical considerations surrounding AI applications in dementia research and care and 2) synthesize these ethical considerations using UNESCO’s four value domains and four basic bioethical principles, with particular attention to implications for diagnosis, management, and dementia care.

## Methods

### Design

This study employed a review of reviews to synthesize evidence from previously published reviews on ethical issues related to AI in dementia care and research. A review of reviews provides a structured synthesis of existing review-level evidence and is particularly appropriate for rapidly evolving fields characterized by conceptual heterogeneity. The protocol of this review is registered in Open Science Framework (https://osf.io/24fd9).

A comprehensive systematic literature search was conducted to identify relevant review articles in accordance with guidance outlined in the Joanna Briggs Institute (JBI) Manual for Evidence Synthesis (Aromataris, 2020). Consistent with JBI recommendations, the search strategy was employed to enhance sensitivity and transparency.

A three-step search strategy was utilized to ensure a comprehensive and systematic literature retrieval. First, a limited initial search was conducted in PubMed and CINAHL to identify relevant articles and to examine keywords in titles and index terms used to describe articles. Second, identified keywords and index terms were used to construct a comprehensive search strategy tailored to each database. The final search was performed on November 21–22^th^ 2025, across the following database: PubMed, CINAHL, IEEE, PsycINFO, and Embase. Other searches such as ProQuest and Google Scholar were included. Boolean operators (AND, OR) were used to combine controlled vocabulary terms (e.g., MeSH) and free-text keywords. The strategy incorporated four primary concept domains: a) artificial intelligence (e.g., “AI,” “large language models,” “ChatGPT,” and “machine learning”), b) dementia (e.g., “Alzheimer’s disease,” “cognitive impairment”), c) ethics (e.g., “ethics,” “autonomy,” and “bias”), and d) caregiver (e.g., “carer,” “family caregiver”). The search strategy was refined following a brief consultation with a biomedical librarian regarding keyword structure and database syntax. Language limits were applied to restrict results to English-language publications. Lastly, the reference lists of all included reviews were screened to identify additional eligible studies. The full, database-specific search terms, including all controlled vocabulary, truncation, and syntax, are provided in Supplementary Appendix A to ensure full reproducibility.

### Inclusion and/or exclusion criteria

Reviews were included if they: 1) addressed populations involving persons living with dementia or their caregivers (Population); 2) examined artificial intelligence technologies such as large language models or conversational systems, or generative AI (Concept); and 3) discussed ethical issues associated with their development, deployment, implementation, or broader implications in dementia care (Context). Eligible studies were restricted to review methodology (e.g., systematic, scoping, integrative, narrative review, or meta-analysis) published in English. Reviews were excluded if they were primary empirical investigations, did not meaningfully engage with ethical dimensions, or lacked an accessible full text.

### Study selection

All identified records were exported in reference management software, Covidence, and duplicates were removed before screening. Titles and abstracts were independently screened by the two authors (E.K. and H.C.) against the eligibility criteria. Full-text articles were subsequently assessed by two authors for final inclusion. Disagreements were resolved through discussion.

**Figure 1:**
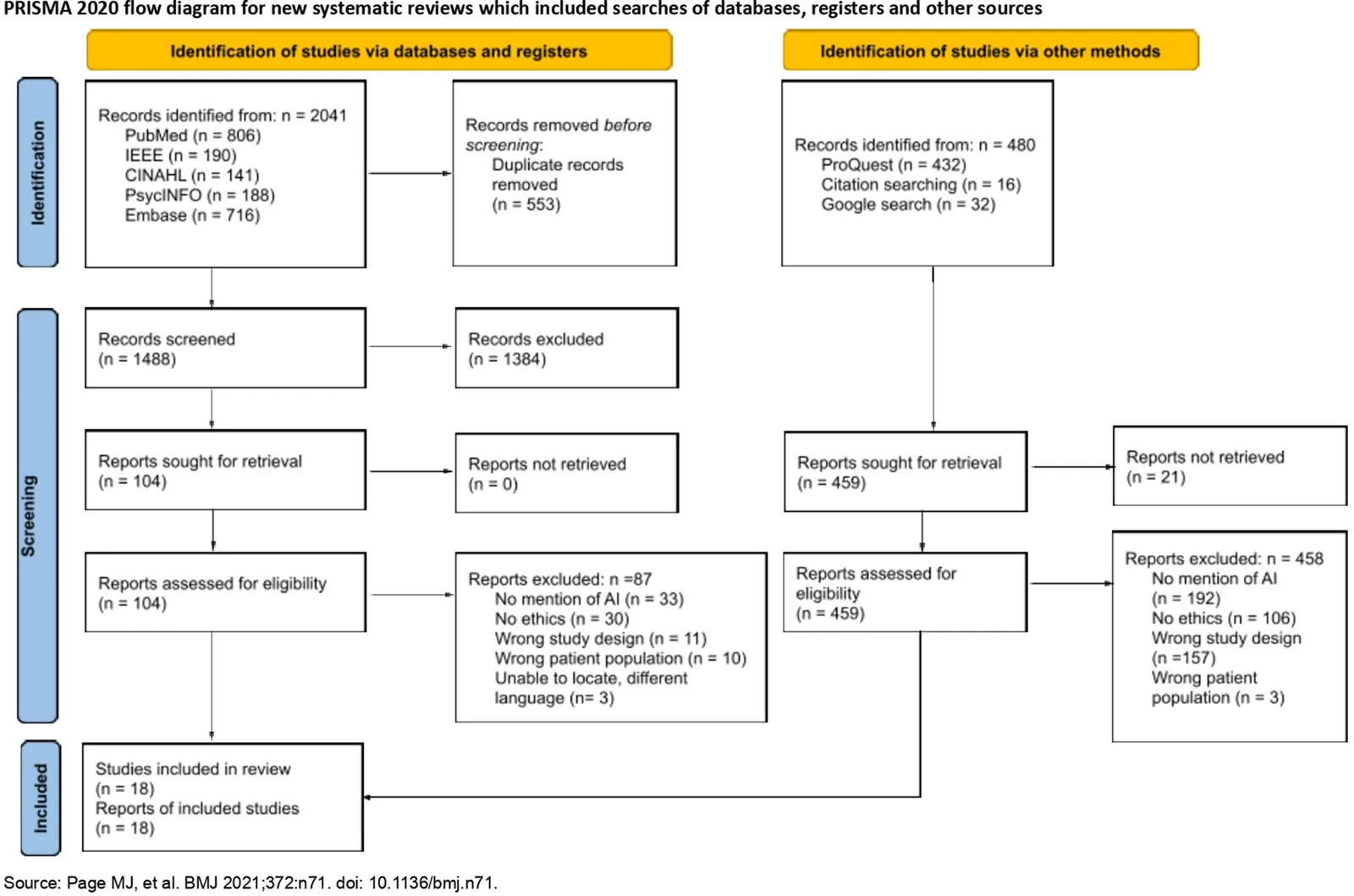
PRISMA 2020 flow diagram for new systematic reviews which included searches of databases, registers, and other sources. *Source*: Page et al. (2021). This work is licensed under CC BY 4.0. To view a copy of this license, visit https://creativecommons.org/licenses/by/4.0/

The study selection process is presented in Figure 1, consistent with the Preferred Reporting Items for Systematic Reviews and Meta-Analyses (PRISMA) flow diagram (Page et al., 2021). The diagram details the number of records identified through database searching, the number of duplicates removed, records screened, full-text articles assessed for eligibility, and the final number of reviews included in the synthesis, along with reasons for full-text exclusions.

### Quality appraisal

The methodological quality and risk of bias of included reviews were rigorously evaluated using a dual-tool approach. First, methodological quality was assessed using the JBI Critical Appraisal Checklist for Systematic Reviews and Research Syntheses (Hilton, 2024). Second, the risk of bias in the review process was evaluated using the Risk of Bias in Systematic Reviews (ROBIS) tool (Whiting et al., 2016). While these appraisal findings were not used as a baseline for excluding studies during the screening phase, they strictly informed data synthesis and the weighting of the evidence.

To evaluate how study quality and potential biases shaped our conclusions, a sensitivity analysis was performed. The included reviews were stratified based on their JBI scores (higher quality ≥ 6 vs lower quality ≤ 5) and ROBIS final judgments (low risk vs high/unclear risk). We subsequently re-evaluated the synthesized themes by temporarily restricting the analysis to 10 higher-quality reviews, while excluding the 8 methodologically weaker reviews, to detect any substantive shifts in our findings. The results demonstrated that the core ethical themes remained consistently unaltered. This allowed us to verify the robustness of our conclusions and ensure that methodologically weak studies did not disproportionately skew the final findings.

### Data abstraction synthesis

A standardized data extraction form was developed a priori. Two authors (E.K. and H.C.) independently extracted data, including authors, year of publication, review type, objectives, population or context, types of AI discussed, ethical domains addressed, key findings, and identified ethical values according to UNESCO guidelines (UNESCO, 2022).

Data were synthesized using a narrative and thematic approach appropriate for a review of reviews synthesizing heterogeneous review designs. Ethical issues were categorized into thematic domains through iterative comparison across included reviews. This synthesis aimed to provide a structure and a critical overview of the current state-of-the-art review-level evidence and to inform gerontology practice, policy development, and future research directions.

A sensitivity analysis was conducted by restricting the synthesis to the 10 higher-quality reviews (i.e., those with a JBI score of 6 or above or a low rating of ROBIS risk of bias) and excluding the 8 methodologically weaker studies. The results demonstrated that the core ethical themes, such as privacy concerns and algorithmic bias, remained consistently unaltered. Consequently, the findings of this study are highly robust and not disproportionately influenced by the methodological quality of the individual source reviews.

## Results

The study selection process was conducted according to PRISMA 2020 guidelines. A total of 2,041 records were identified through database searches. Separately, an additional 480 records were identified through other sources, including ProQuest (*n* = 432), Google search (*n* = 32), and citation searching (*n* = 16). For the database registry, after removing 1,384 records, 1,488 records remained for title and abstract screening; of these, 1,384 records were excluded. Subsequently, 104 full-text reports were retrieved and assessed for eligibility. After full-text review, 87 reports were excluded due to the absence of artificial intelligence content (*n* = 33), lack of ethical relevance (*n* = 30), inappropriate study design (*n* = 11), incorrect patient population (*n* = 10), or inability to retrieve the article because of language-related limitations (*n* = 3). For the other sources, 459 reports were sought for retrieval and assessed for eligibility, resulting in the inclusion of 1 study. Ultimately, a total of 18 individual reviews were included in this review of reviews (see Table 1).

**Table 1:** Study characteristics.

Author, year	Country	Study purpose	Study Design	Search databases for review	Total number of studies	Target Chronic Condition	AI-based methods and algorithms	Device (medium/ modalities of AI)
**Bhuiyan et al. (2025)**	Bangladesh	To synthesize evidence on "smart neuronutrition" (intersection of nutrition, neuroscience, and AI) to optimize brain function and cognitive resilience	Critical review	N/R	N/R	Neurodegenerative disorders (AD, Parkinson’s disease)	Machine learning, deep learning, natural language processing, computer vision	Wearable biosensors, mobile apps (e.g., goFOOD), 3D food printers, image-based dietary tracking
**de Aranha Martins (2021)**	Portugal	To explore ethical issues of using IAT in people living with dementia.	Systematic review and expert interviews	Google Scholar	67	Dementia	IAT (Robotics, automated decision‐making systems, ML)	Social assistive robots, smart home monitoring, virtual assistants
**El-Sappagh et al. (2023)**	Egypt, South Korea, Spain	To review AI systems for Alzheimer’s diagnosis and progression, focusing on Trustworthy AI requirements	Systematic review	PubMed, ScienceDirect, SpringerLink, Nature, IEEE Xplore, Web of Science, Scopus, arXiv, medRxiv	110 papers were included (86 papers did not explicitly take care of trustworthy AI)	AD, MCI	Supervised machine learning (support vector machines, decision trees), deep learning (convolutional neural network, recurrent neural network), ensembles	Clinical decision support systems, neuroimaging tools (MRI, PET)
**Ford et al. (2023)**	The United Kingdom	To assess digital biomarkers and AI for early dementia detection against ethical screening principles	Conceptual/ ethical review	N/R	N/R	Dementia and AD	Supervised learning, unsupervised learning	Wearables (smartwatches), Smartphones, In-home/In-car sensors, EHR data
**Graham et al. (2020)**	USA	To provide a primer for clinicians on the understanding and use of an exciting new approach to supporting clinical decision-making such as diagnosis, prediction, and differentiation between the various types of MCI and dementias – i.e., AI	Conceptual review	N/R	21	Cognitive decline in older adults	Machine learning (supervised, unsupervised, semi-supervised, deep learning), natural language processing (speech recognition, sentiment analysis, optical character recognition)	Clinical and psychometric assessments, neuroimaging and neurophysiologic data, EHR and claims data, converting spoken words to data, extracting opinions and affecting from text, converting written and printed text into data
**Harrison et al. (2025)**	The United Kingdom	To explore the prospect of large language models transforming dementia diagnosis and care, while addressing ethical and practical considerations	Narrative Review	References scientific literature and pre-print servers (e.g., arXiv) and external datasets (e.g., ADNI)	N/R	Dementia	Machine learning (support vector machines, random forest, convolutional neural networks), natural language processing, large language models	Neuroimaging (MRI, FDG-PET), CSF biomarkers, genetics, computer/software interface, ambient voice recording, integration with EHR
**Hassan et al. (2025)**	Egypt	To evaluate how the convergence of AI and nanotechnology can enable a shift from reactive to predictive neuromedicine for neurodegenerative diseases	Narrative review	PubMed, Scopus, and Web of Science	N/R	Neurodegenerative diseases (AD, Parkinson’s, Amyotrophic lateral sclerosis, Huntington’s, multiple sclerosis)	Machine learning, deep learning (generative adversarial networks, graph neural networks, reinforcement learning)	Nanodiagnostics, nanosensors, wearables (e.g., smartwatches, EEG headbands), Digital Twins, theranostic nanoplatforms
**Hcini et al. (2024)**	Saudi Arabia	To provide a comprehensive review of deep learning techniques for AD classification using brain imaging	Comprehensive Review	Springer, IEEE Xplore, ScienceDirect, MDPI, Elsevier, Wiley, ACM, ADNI, OASIS	61	AD	Deep learning (convolutional neural network, Vision Transformers), hybrid AI	Neuroimaging
**Hernández-Torres and Sánchez-DelaCruz (2025)**	Mexico	To analyze the evolution, challenges, and opportunities of Ambient Intelligence in the 21st century	Systematic Review	Scopus, Web of Science, Google Scholar	38	General health monitoring including dementia and chronic diseases	Supervised machine learning (support vector machines, decision trees, K-nearest neighbors, Naïve Bayes), deep learning (artificial neural network), Fuzzy Logic	IoT sensors, wearables, smart homes, ambient intelligence environments
**Hine et al. (2022)**	The United Kingdom	To explore ethical issues in the design and implementation of home-based smart care/remote monitoring for people living with dementia	Conceptual review/ case study	N/R	N/R	Dementia	Machine learning (federated learning), pattern recognition algorithms	IoT sensors (smart plugs, passive infrared movement sensors, door sensors), Physiological monitors, Dashboard
**Jiao (2025)**	The United Kingdom	To examine music therapy and brainwave entrainment and propose an integrative framework using AI-driven biofeedback for personalized intervention	Mini review	PubMed, Scopus, Web of Science, Google Scholar	N/R	Mental health disorders, Cognitive decline (e.g., AD)	Machine learning, AI-driven biofeedback	Wearable sensors, EEG monitors, mobile apps, Brainwave entrainment (binaural beats/isochronic tones)
**Kale et al. (2024)**	India	To explore recent advancements in AI applications across various stages of AD management	Review (not specified)	N/R	N/R	AD	Machine learning (support vector machines, random forest, multi-feature kernel discriminant dictionary learning, logistic regression), deep learning (convolutional neural network, non-convolutional artificial neural network)	Neuroimaging (MRI, CT, PET), biomarker identification (blood tests and cerebrospinal fluid analyses), AI-enhanced neuropsychological tests, and tools for speech and language changes
**Lai et al. (2026)**	Canada	To examine technologies used for detecting behavioral anomalies, the activities they monitor, and the trade-offs between their benefits and limitations	Narrative review using a thematic analysis	Medline, IEEE Xplore, ACM Digital Library, and Web of Science	78	Dementia	Deep learning (convolutional neural networks, recurrent neural networks, long short-term memory networks, autoencoders), unsupervised learning (clustering), hybrid AI	GPS-based tracking systems, wearable technologies, environmental sensors, smart home and IoT systems, audio and video monitoring systems, computer-based and digital monitoring
**Rohrer et al. (2025)**	Switzerland	To provide the reader with an overview of machine learning techniques frequently used in dementia therapy	Systematic review	ACM Digital Library, IEEE Xplore, ACL Anthology, PubMed, Springer Link, Google Scholar	23	AD and cognitive impairment	Deep learning (convolutional neural network, long short-term memory, Transformer, Encoder and Decoder), reinforcement learning (Deep Q-network, soft actor-critic, asynchronous advantage actor critic), hybrid AI, recommendation system, rule-based system	Recommendation systems, smartphone/tablet, laptop, robot, virtual reality, personal computer and Kinect camera
**Rudroff et al. (2024)**	USA, Finland	To summarize the current state of AI applications in neuroimaging for early AD prediction and to highlight the potential of AI techniques in improving early AD diagnosis, prognosis, and management	Narrative review	N/R	N/R	AD	Machine learning, deep learning (convolutional neural networks, recurrent neural networks, autoencoder), hybrid AI	These methods have been successfully applied to various neuroimaging modalities, including structural MRI, fMRI, and PET imaging, to classify different stages of AD
**Sætra (2022)**	Norway	To discuss the ethics of social robots and how we can take a principled approach to when and how we employ them	Review and cross-sectional quantitative study design	No information about the review; questionnaire for the quantitative study design	N/R	Dementia	Large language models	Application Programming Interface using tablet, laptop/personal computer, smartphone, which is run in a data center and via an internet connection
**Salvi et al. (2025)**	USA, Malta	To present a comprehensive analysis of IoT-enabled systems tailored to AD care, focusing on wearable biosensors, cognitive monitoring tools, smart home automation, and AI-driven analytics.	Systematic review	IEEE Xplore, PubMed, Scopus, Web of Science, ACM Digital Library, and Google Scholar	236	AD	Supervised machine learning (support vector machines, random forest, XGBoost), deep learning (convolutional neural network, deep residual network-long short-term memory network)	Pace, rhythm, and variability features; cortical region of interest features, raw fMRI volumes, audio, video, and motion sensors
**Ursin et al. (2021)**	Germany	To explore the ethical arguments put forward for AI-aided AD prediction in subjectively asymptomatic individuals and their ethical implications	Systematic review using a thematic analysis	PubMed, Cochrane, PhillPapers	18	AD prediction of asymptomatic individuals	N/R	N/A

*Note.* IAT = intelligence assistive technology; AI = artificial intelligence; AD = Alzheimer’s disease; EEG = electroencephalogram; EHR = electronic health record; IoT = internet of things; MCI = mild cognitive impairment; N/R = not reported.

### Study characteristics

Most of the included reviews were published between 2023 and 2025, except for five (de Aranha Martins, 2021; Graham et al., 2020; Hine et al., 2022; Sætra, 2022; Ursin et al., 2021). Countries in which the authors conducted the studies varied; the countries with the highest number of studies were: four reviews from the UK (Ford et al., 2023; Harrison et al., 2025; Hine et al., 2022; Jiao, 2025) and three from the USA (Graham et al., 2020; Rudroff et al., 2024; Salvi et al., 2025). Most reviews focused on AI applications for diagnosis and early prediction of Alzheimer’s disease (El-Sappagh et al., 2023; Ford et al., 2023; Graham et al., 2020; Harrison et al., 2025; Hassan et al., 2025; Hcini et al., 2024; Kale et al., 2024; Lai et al., 2026; Rudroff et al., 2024; Salvi et al., 2025; Ursin et al., 2021).

To examine the study objectives, the authors used systematic review designs the most (*n* = 6) (de Aranha Martins, 2021;  El-Sappagh et al., 2023; Hernández-Torres & Sánchez-DelaCruz, 2025; Rohrer et al., 2025; Salvi et al., 2025; Ursin et al., 2021), followed by narrative reviews (*n* = 4) (Harrison et al., 2025; Hassan et al., 2025; Lai et al., 2026; Rudroff et al., 2024). Half of the included reviews reported the number of studies that they included (*n* = 9), ranging from 18 (Ursin et al., 2021) to 236 studies (Salvi et al., 2025). Particularly, non-systematic reviews (Bhuiyan et al., 2025; Ford et al., 2023; Harrison et al., 2025; Hassan et al., 2025; Hine et al., 2022; Jiao, 2025; Kale et al., 2024; Rudroff et al., 2024; Sætra, 2022) did not report the number of included studies. Most studies particularly focused on Alzheimer’s disease (Bhuiyan et al., 2025; Hcini et al., 2024; Kale et al., 2024; Rudroff et al., 2024; Salvi et al., 2025; Ursin et al., 2021).

### AI-based applications identified in dementia care

Various AI-based methods were employed, mostly involving machine learning and deep learning techniques (see Table 1). Machine learning methods such as support vector machines and random forests have been widely used to classify clinical or behavioral patterns associated with cognitive decline. For instance, a systematic review of 38 studies (Hernández-Torres & Sánchez-DelaCruz, 2025) reported the use of supervised machine learning algorithms—such as decision trees, naïve Bayes, k-nearest neighbors, and support vector machines—to support ambient intelligence systems for applications including remote monitoring of chronic patients and behavior prediction in dementia care. In contrast, deep learning methods such as convolutional neural networks (CNNs) can automatically extract complex features from high- dimensional data (e.g., neuroimaging) to support disease detection and prediction (El-Sappagh et al., 2023).

Some reviews employed natural language processing and large language models (*n* = 5, Bhuiyan et al., 2025; Ford et al., 2023; Graham et al., 2020; Harrison et al., 2025; Sætra, 2022), while four studies reported hybrid models combining multiple AI techniques (*n* = 4, Hcini et al., 2024; Kale et al., 2024; Lai et al., 2026; Rohrer et al., 2025). Hybrid models often combine deep learning and machine learning approaches, where deep learning is used for feature extraction and machine learning algorithms are applied for classification. For example, in anomaly detection systems for dementia care, CNNs analyze video data to detect behaviors such as wandering or agitation, while recurrent neural networks (RNNs) are used to analyze time-series sensor data to identify deviations in daily movement or routines (Lai et al., 2026).

### Ethical concerns across dementia care

Table 2 represents the ethical concerns across dementia care across the reviewed studies. Ethical considerations in AI for dementia diagnosis and assessment involved algorithmic bias, limited data diversity, transparency, explainability, accuracy, autonomy, and consent. Several studies reported concerns that limited or unrepresentative datasets may reduce diversity and generalizability and cause algorithmic biases across race, gender, and other underrepresented groups (Bhuiyan et al., 2025; El-Sappagh et al., 2023; Harrison et al., 2025; Hassan et al., 2025; Hcini et al., 2024; Hernández-Torres & Sánchez-DelaCruz, 2025; Jiao, 2025; Kale et al., 2024; Rudroff et al., 2024; Salvi et al., 2025). Transparency, explainability, and communication about AI use and limitations were also frequently identified as important for trust and responsible AI-supported decision-making (Bhuiyan et al., 2025; El-Sappagh et al., 2023; Ford et al., 2023; Graham et al., 2020; Hassan et al., 2025; Hcini et al., 2024; Jiao, 2025; Kale et al., 2024; Rudroff et al., 2024). Other concerns included accuracy, fake or synthetic data, informed consent, and the ethical tension between knowing and not knowing dementia risk information (de Aranha Martins 2021; Ford et al., 2023; Harrison et al., 2025; Hcini et al., 2024; Hernández-Torres & Sánchez-DelaCruz, 2025; Hine et al., 2022; Lai et al., 2026; Rohrer et al., 2025; Sætra, 2022; Ursin et al., 2021).

**Table 2:** Overview of ethical concerns across dementia care.

Author et al. (year)	AI ethical considerations across dementia care
Bhuiyan et al. (2025)	1) Diagnosis/assessment • Algorithmic bias and limited data about diverse racial, ethnic, rural, older, or low-income populations. • Lack of transparency and explainability as barriers to clinical trust, patient understanding, and user autonomy. 2) Management/Treatment • Privacy and security risks involving sensitive dietary, genomic, and health-related data. • Fragmented regulatory oversight, especially for AI-guided tools that may bypass rigorous medical-device evaluation. • Digital divide concerns related to AI tools that assume access to smartphones, the internet, or digital literacy.
de Aranha Martins (2021)	1) Diagnosis/assessment • Algorithmic bias. • Transparency and accessibility. • Autonomy and stigma. • Informed use. 2) Management/treatment • Privacy and surveillance risks from robots or AI systems collecting data in private care spaces. • Security vulnerabilities, hacking risks, and legal responsibility in AI and robotic dementia care. • Using AI unethically and under human control. • The right to an “authentic self” life. • Safety and trust. • Human well-being, emotional, social, and financial harm. • Accountability and responsibility. • Potential environmental harm while using AI. • Concerns about managing and progressing advanced machine learning and appropriate regulation. 3) Caregiving • Deception and authenticity concerns related to the device that appear to express emotions, including false intimacy or confusion for people with dementia. • Infantilization risks in older adult care, including potential threats to dignity and respect. • Technology-centered care and possible reduction of human contact, with increased risk of social isolation.
El-Sappagh et al. (2023)	1) Diagnosis/assessment • Support for the decision-making of physicians and patients without replacing them or subjecting them to fully automated decisions. • Transparency, traceability, explainability, and communication regarding the use and limitations of AI. • Diversity and algorithmic biases. 2) Management/treatment • Privacy and data protection. • Quality and data integrity. • Respect for autonomy based on the consideration of human rights, human-involved decisions, and human oversight. • Data governance, access to data, accountability, and stakeholder participation. • Overreliance on AI. • Fairness. • Technical robustness and safety, security, accuracy, reliability, and reproducibility. • Societal and environmental well-being.
Ford et al. (2023)	1) Diagnosis/assessment • Limited explainability and accuracy of dementia detection models. • False positives generated by widespread AI. • Transparency in overriding AI outputs and explaining black-box recommendations. • Algorithmic bias and health inequalities from models trained on unrepresentative cohorts, such as wealthy or predominantly White samples. • The value of knowing or not knowing test results and their impact on an individual’s future. 2) Management/treatment • Potential invasion of privacy. • The existence of a digital divide. • Accountability and fairness regarding the error liability.
Graham et al. (2020)	1) Diagnosis/assessment • Bias and generalizability problems from the algorithms developed. • Challenge of transparency and building trust in the model ("black box" of machine learning). • Need for developing explainable AI for communication between clinicians and patients and their families. 2) Management/treatment • User privacy and surveillance risks in scalable AI tools. • Data ownership.
Harrison et al. (2025)	1) Diagnosis/assessment • Transparency and explainability needs due to black-box systems, verification difficulties, and accountability challenges. • Racial and gender bias embedded in training data and model outputs. • Algorithmic bias in race and gender. • Hallucination risks in models, including confident but incorrect information. 2) Management/treatment • Privacy in sensitive fields (e.g., treatments). • Human-in-the-loop oversight, factual verification, references, and continuous monitoring for harm reduction. • Patient autonomy, the right to data control. • Fairness. • Accountability.
Hassan et al. (2025)	1) Diagnosis/assessment • Algorithmic bias and skewed datasets as sources of reinforced health inequities. • Transparency concerns regarding black-box AI systems, limited clinical interpretation, and reduced trust in AI-supported decisions. 2) Management/treatment • Privacy, data ownership, and consent concerns involving sensitive data such as images and genomic information. • Cybersecurity. • Regulatory gaps for advanced or hybrid AI systems. • Economic and global inequities, including limited accessibility of costly technologies in low- and middle-income settings.
Hcini et al. (2024)	1) Diagnosis/assessment • Transparency, explainability, and interpretability problems in deep learning models, especially limited clinician understanding of prediction bases. • Dataset imbalance and lack of diversity as sources of bias and limited generalizability. • Potential technological abuse by the clarification of Alzheimer’s images. 2) Management/treatment • Privacy concerns involving sensitive patient data. • Fairness based on ethical and social issues and technical and computational limitations. • Need for strict data protection, confidentiality requirements, and safeguards.
Hernández-Torres and Sánchez-DelaCruz (2025)	1) Diagnosis/assessment • Algorithmic bias from imbalanced training data. • Transparency and security. • Autonomy concerns with passive data provision. 2) Management/treatment • Privacy and surveillance risks from continuous, real-time data collection that may compromise anonymity. • A lack of trust regarding privacy or the data complexity. • Cybersecurity risks, including data poisoning, spoofing, and cyber-physical attacks. • Need for strengthened regulatory frameworks.
Hine et al. (2022)	1) Diagnosis/assessment • Transparency issue. • Accuracy, potential false positives. • Dignity concern; need for preservation of autonomy. • Consent. 2) Management/treatment • Privacy, acceptability, and security. • Representativeness of training data for fairness. • Digital divide due to unequal algorithmic literacy. • Need for personalized system approaches to ensure patients and carers' well-being. 3) Caregiving • Reduction of care to “sensing and measuring,” with potential weakening of relational and human-centered care.
Jiao (2025)	1) Diagnosis/assessment • Algorithmic bias from underrepresentation in training datasets and potential unequal benefits. • Transparent algorithm design and routine auditing as safeguards for responsible AI use. 2) Management/treatment • Need for protection of data privacy. • Data governance, informed consent, and secure handling of sensitive health data.
Kale et al. (2024)	1) Diagnosis/assessment • Transparency in algorithmic decision-making. • Limited large and diverse datasets, reduced generalizability, and performance limitations across patient groups. 2) Management/treatment • Privacy and cybersecurity concerns, including robust data protection, encryption, and regulatory compliance. • Informed consent for AI-related data use. • Intellectual property, regulatory approval, and interdisciplinary clinical integration. • Equity in access to AI-enabled approaches in healthcare.
Lai et al. (2026)	1) Diagnosis/assessment • False positives, handling complex data with calibration to maintain accuracy. • Need for large, high-quality datasets. • Data reliability. 2) Management/treatment • Need to preserve privacy; balancing intrusiveness with proper monitoring.
Rohrer et al. (2025)	1) Diagnosis/assessment • Fake or synthetic data concerns in AI-related research. • Consent issues with patients having advanced stages of the disease. • System reliability. 2) Management/treatment • Responsibility and accountability.
Rudroff et al. (2024)	1) Diagnosis/assessment • Transparency and explainability issues; need for interpretable and explainable AI. • The limited availability of open access data, especially large, independent, diverse validation datasets, limiting generalizability and robustness of AI. 2) Management/treatment • Privacy, security, and informed consent. • Need for regulatory approval for data privacy and security, liability, and user acceptance to integrate AI into clinical practice. • Need for standardized protocols and data preprocessing for validity and comparability.
Sætra (2022)	1) Diagnosis/assessment • Dignity and respect. 2) Caregiving • Deception that makes humans believe that the systems are real and infantilization of users. • Tendencies to overly reduce human contact.
Salvi et al. (2025)	1) Diagnosis/assessment • Limited multicenter and longitudinal datasets as barriers to generalizability and external validity. • Small samples, modality silos, and lack of shared benchmarks as causes of inflated performance estimates. • Transparency. • Potential inequitable performance across groups due to demographic underrepresentation. • Insufficient explainability and clinician usability evidence as barriers to adoption in clinical workflows. • Latency and reliability variability due to integration complexity. 2) Management/treatment • Privacy, security, and governance weaknesses that may reduce stakeholder trust and increase misuse risks. • Cost and resource constraints, which affect continuous monitoring and scalability. • Lack of standardized, validated evaluation tools; uncertain regulatory pathway. • Fairness, responsibility, and privacy yet under-represent multi-level sociological impacts. • Need to ensure the robustness of AI models.
Ursin et al. (2021)	1) Diagnosis/assessment • Transparency and explicability, uncertainty of risk assessment. • Algorithmic bias. • Accuracy concerns, including false positives, false negatives, insufficient predictive power, and difficulty communicating probabilistic risk. • Ethical tension between the right to know and the right not to know one’s dementia risk, respect for autonomy and empowerment, good communication of a physician for disclosure, clarity, informing family members. • Cost-effectiveness and restriction of tests. • Consent for diagnosis. 2) Management/treatment • Data privacy. • Psychological and social harms (e.g., anxiety, uncertainty, stigma, health insurance discrimination, and employment discrimination). • Concerns about data governance, accountability, passive surveillance, and regulatory approval issues. • A need for different liability models for different use cases and for a risk liability system. • Need for physician training, disclosure guidelines, liability models, and standardization of test methods and values.

*Note.* AI = artificial intelligence.

For management and treatment, articles generally reported privacy, data governance, equitable access to the system or digital divide, and well-being and harms as ethical concerns. Privacy, security, and data protection concerns include sensitive data use, privacy invasion, safety, surveillance, informed consent of data use for AI, cybersecurity risks, secure data handling, and strict data protection (Bhuiyan et al., 2025; de Aranha Martins, 2021; El-Sappagh et al., 2023; Ford et al., 2023; Graham et al., 2020; Harrison et al., 2025; Hassan et al., 2025; Hcini et al., 2024; Hernández-Torres & Sánchez-DelaCruz, 2025; Hine et al., 2022; Jiao, 2025; Kale et al., 2024; Lai et al., 2026; Rudroff et al., 2024; Salvi et al., 2025; Ursin et al., 2021). Data management and regulation concerns include accountability, fragmented regulatory oversight, liability, standardized protocols, validated evaluation tools, system reliability, and the need to ensure robust AI models (Bhuiyan et al., 2025; de Aranha Martins, 2021; El-Sappagh et al., 2023; Ford et al., 2023; Graham et al., 2020; Harrison et al., 2025; Hassan et al., 2025; Hernández-Torres & Sánchez-DelaCruz, 2025; Jiao, 2025; Kale et al., 2024; Rohrer et al., 2025; Rudroff et al., 2024; Salvi et al., 2025; Ursin et al., 2021).

Additionally, fairness and inequity concerns include the digital divide, limited access to costly technologies, and economic or global inequities in AI-enabled care (Bhuiyan et al., 2025; de Aranha Martins, 2021; El-Sappagh et al., 2023; Ford et al., 2023; Harrison et al., 2025; Hassan et al., 2025; Hcini et al., 2024; Hine et al., 2022; Kale et al., 2024; Salvi et al., 2025). Finally, other ethical concerns include patient autonomy, overreliance on AI, safety and trust, human well-being, emotional, social, and financial harm, and potential environmental harm (de Aranha Martins 2021; El-Sappagh et al., 2023; Hine et al., 2022; Ursin et al., 2021). Meanwhile, caregiving-related ethical issues included deception and authenticity concerns, infantilization, reduced human contact, social isolation, technology-centered care, and possible weakening of relational or human-centered care (de Aranha Martins, 2021; Hine et al., 2022; Sætra, 2022).

### Identified themes based on UNESCO AI value domains

Table 3 presents the themes and categories from the included reviews. To systematically examine how ethical concerns were framed across the included reviews, we used the four AI-related value domains of the UNESCO Recommendation on the Ethics of Artificial Intelligence (UNESCO, 2022). These include: 1) respect, protection, and promotion of human rights and fundamental freedoms and human dignity, 2) environment and ecosystem flourishing, 3) ensuring diversity and inclusiveness, and 4) living in a peaceful, just, and interconnected society. When categorized into the four AI-related value domains, most ethical concerns fell under respect for human rights and dignity, and diversity and inclusiveness.

**Table 3:** Artificial intelligence value domains from UNESCO (UNESCO, 2021) and identified themes and categories.

UNESCO AI values	Themes (the number of articles)	Categories	Key points and supporting references	Related bioethical principles
**Respect, protection, and promotion of human rights and fundamental freedoms and human dignity**	Protection of human rights (n = 17)	Privacy	Privacy of sensitive health data and privacy intrusiveness (Bhuiyan et al., 2025; de Aranha Martins, 2021; El-Sappagh et al., 2023; Ford et al., 2023; Graham et al., 2020; Harrison et al., 2025; Hassan et al., 2025; Hcini et al., 2024; Hernández-Torres & Sánchez-DelaCruz, 2025; Hine et al., 2022; Jiao, 2025; Kale et al., 2024; Lai et al., 2026; Rudroff et al., 2024; Salvi et al., 2025; Ursin et al., 2021)	- Respect for autonomy - Non-maleficence
		Informed consent	Informed consent for data use, voluntariness (de Aranha Martins, 2021; Hassan et al., 2025; Hine et al., 2022; Jiao, 2025; Kale et al., 2024; Rohrer et al., 2025; Rudroff et al., 2024; Ursin et al., 2021)	− Non-maleficence
		Security	Data security and protection (de Aranha Martins, 2021; El-Sappagh et al., 2023; Hassan et al., 2025; Hcini et al., 2024; Hernández-Torres & Sánchez-DelaCruz, 2025; Hine et al., 2022; Jiao, 2025; Kale et al., 2024; Lai et al., 2026; Rudroff et al., 2024; Salvi et al., 2025)	− Non-maleficence
		Malicious use	Malicious use, potential technological abuse, and ethical misuse of AI (de Aranha Martins, 2021; Hcini et al., 2024)	− Respect for autonomy
	Technical trustworthiness (n = 17)	Transparency	Black box problem, explicability (Bhuiyan et al., 2025; de Aranha Martins, 2021; El-Sappagh et al., 2023; Ford et al., 2023; Graham et al., 2020; Harrison et al., 2025; Hassan et al., 2025; Hcini et al., 2024; Hernández-Torres & Sánchez-DelaCruz, 2025; Hine et al., 2022; Jiao, 2025; Kale et al., 2024; Rudroff et al., 2024; Salvi et al., 2025; Ursin et al., 2021)	- Respect for autonomy - Beneficence
		Explainability	Lack of explainability and interpretability (Bhuiyan et al., 2025; El-Sappagh et al., 2023; Ford et al., 2023; Graham et al., 2020; Harrison et al., 2025; Hassan et al., 2025; Hcini et al., 2024; Rudroff et al., 2024; Salvi et al., 2025; Ursin et al., 2021)	- Respect for autonomy - Beneficence
		Accuracy	Prediction accuracy uncertainty, false positives or negatives, hallucination, clinical relevance, need for validation, potential use of fake data (El-Sappagh et al., 2023; Ford et al., 2023; Harrison et al., 2025; Hine et al., 2022; Lai et al., 2026; Rohrer et al., 2025; Ursin et al., 2021)	− Beneficence
		Reliability	System reliability variability, data integrity (El-Sappagh et al., 2023; Lai et al., 2026; Rohrer et al., 2025; Salvi et al., 2025)	− Beneficence
		Robustness	Limited scrutiny, technical robustness and safety (El-Sappagh et al., 2023; Rudroff et al., 2024; Salvi et al., 2025)	− Beneficence
	Respect for human dignity (n = 10)	Autonomy	Autonomy, the right of living an authentic life, the right to data control, dignity in care (Bhuiyan et al., 2025; de Aranha Martins, 2021; El-Sappagh et al., 2023; Harrison et al., 2025; Hernández-Torres & Sánchez-DelaCruz, 2025; Hine et al., 2022; Sætra, 2022; Ursin et al., 2021)	− Respect for autonomy
		Trust	Challenges of establishing or improving trust (Bhuiyan et al., 2025; de Aranha Martins, 2021; Graham et al., 2020; Hernández-Torres & Sánchez-DelaCruz, 2025)	− Beneficence
		Psycho-socioeconomic well-being	Psychological harm (stress, anxiety, stigmatization), family burden, financial harm, social harm and well-being, fear of technology-centered care (de Aranha Martins, 2021; El-Sappagh et al., 2023; Hine et al., 2022; Ursin et al., 2021)	- Respect for autonomy - Beneficence
		Moral decision- making	Right to know vs right not to know, challenging to make moral decisions (Ford et al., 2023; Ursin et al., 2021)	− Non-maleficence
		Impersonal care	Deception, authenticity, infantilization; the absence of relational care among caregiving dyads (de Aranha Martins, 2021; Hine et al., 2022; Sætra, 2022)	− Non-maleficence
		Reliance on AI	Overreliance on AI with uncritical deployment (El-Sappagh et al., 2023)	− Non-maleficence
**Environment and ecosystem flourishing**	Environmental well-being (n = 2)	Environmental well-being	Environmental harm, sustainability, environmental friendliness (de Aranha Martins, 2021; El-Sappagh et al., 2023)	N/A
**Ensuring diversity and inclusiveness**	Equity and Access (n = 16)	Dataset diversity	Lack of diversity in datasets, limited availability of large/high-quality datasets (Bhuiyan et al., 2025; El-Sappagh et al., 2023; Hcini et al., 2024; Kale et al., 2024; Lai et al., 2026; Rudroff et al., 2024; Salvi et al., 2025)	− Justice
		Algorithmic bias	Race, gender, cultural and linguistic bias (Bhuiyan et al., 2025; de Aranha Martins, 2021; El-Sappagh et al., 2023; Ford et al., 2023; Graham et al., 2020; Harrison et al., 2025; Hassan et al., 2025; Hernández-Torres & Sánchez-DelaCruz, 2025; Jiao, 2025; Ursin et al., 2021)	− Justice
		Fairness	Fairness, representativeness (El-Sappagh et al., 2023; Harrison et al., 2025; Hassan et al., 2025; Hcini et al., 2024; Hine et al., 2022; Rudroff et al., 2024)	− Justice
		Digital divide	Digital health access barriers (Bhuiyan et al., 2025; Ford et al., 2023; Hassan et al., 2025; Hine et al., 2022)	− Justice
		Structural inequities	Health inequities particularly among economically and geographically underserved populations (Ford et al., 2023; Hassan et al., 2025; Kale et al., 2024)	− Justice
		Cost & resources	Cost inequality and resource constraints (Salvi et al., 2025; Ursin et al., 2021)	− Justice
**Living in a peaceful, just, and interconnected society**	Governance (n = 15)	Regulatory oversight	Regulatory frameworks and approval processes, fragmented regulatory oversight, limited validation of tools, interoperability gaps, redefining clinical roles, lawfulness, limited risk stratification, need for establishing common AI-related protocols (Bhuiyan et al., 2025; de Aranha Martins, 2021; El-Sappagh et al., 2023; Harrison et al., 2025; Hassan et al., 2025; Hernández-Torres & Sánchez-DelaCruz, 2025; Jiao, 2025; Kale et al., 2024; Lai et al., 2026; Rudroff et al., 2024; Salvi et al., 2025; Ursin et al., 2021)	− Non-maleficence
		Accountability	Liability complexity, responsibility, data ownership, need for ethical framework and stakeholder engagement, human-centered AI implementation (de Aranha Martins, 2021; El-Sappagh et al., 2023; Ford et al., 2023; Graham et al., 2020; Harrison et al., 2025; Rohrer et al., 2025; Salvi et al., 2025; Ursin et al., 2021)	− Justice
		Intellectual property	Intellectual property rights (Kale et al., 2024)	N/A

*Note.* N/A = not applicable.

#### Respect, protection, promotion of human rights, fundamental freedoms, and human dignity

This domain is about whether AI systems respect and protect human dignity, autonomy, and fundamental rights (UNESCO, 2022). Under this domain, three themes were identified: 1) protection of human rights, 2) technical trustworthiness, and 3) respect for human dignity.

##### Protection of human rights

All included reviews but one discussed concerns about the protection of human rights, particularly the privacy of sensitive health data (Bhuiyan et al., 2025; de Aranha Martins, 2021; El-Sappagh et al., 2023; Ford et al., 2023; Graham et al., 2020; Harrison et al., 2025; Hassan et al., 2025; Hcini et al., 2024; Hernández-Torres & Sánchez-DelaCruz, 2025; Hine et al., 2022; Jiao, 2025; Kale et al., 2024; Lai et al., 2026; Rudroff et al., 2024; Salvi et al., 2025; Ursin et al., 2021). Informed consent in progressive cognitive decline was reported as a critical ethical issue while using AI systems (de Aranha Martins, 2021; Hassan et al., 2025; Hine et al., 2022; Jiao, 2025; Kale et al., 2024; Rohrer et al., 2025; Rudroff et al., 2024; Ursin et al., 2021). Risks of data security and protection (de Aranha Martins, 2021; El-Sappagh et al., 2023; Hassan et al., 2025; Hcini et al., 2024; Hernández-Torres & Sánchez-DelaCruz, 2025; Hine et al., 2022; Jiao, 2025; Kale et al., 2024; Lai et al., 2026; Rudroff et al., 2024; Salvi et al., 2025) and malicious use (de Aranha Martins, 2021; Hcini et al, 2024) have raised further concerns in long-term dementia care settings, where continuous monitoring is often necessary and protection against dangerous or inaccurate information is essential.

##### Technical trustworthiness

Another theme about ethical concern under this UNESCO domain was technical trustworthiness, referring to how trustworthy an algorithm is and using the right technical measures to evaluate it, including reliability and understandability (Paramonova et al., 2024). A total of 17 reviews reported these ethical concerns. Technical issues such as a lack of transparency, limited explainability, prediction uncertainty, reliability variability, weak data integrity, and limited robustness not only limited performance but also affected individuals’ rights to receive accurate and trustworthy care. One of the most common issues was the transparency of AI systems, often described as the “black box” problem (Graham et al., 2020; Harrison et al., 2025; Hassan et al., 2025; Ursin et al., 2021). This reduced transparency particularly in diagnosis and assessment and complicated the accountability (Bhuiyan et al., 2025; de Aranha Martins, 2021; El-Sappagh et al., 2023; Ford et al., 2023; Graham et al., 2020; Harrison et al., 2025; Hassan et al., 2025; Hcini et al., 2024; Hernández-Torres & Sánchez-DelaCruz, 2025; Hine et al., 2022; Jiao, 2025; Kale et al., 2024; Rudroff et al., 2024; Salvi et al., 2025; Ursin et al., 2021).

Insufficient explainability and interpretability were also identified as barriers that limited the capacity to understand and critically evaluate AI-based decisions (Bhuiyan et al., 2025; El-Sappagh et al., 2023; Ford et al., 2023; Graham et al., 2020; Harrison et al., 2025; Hassan et al., 2025; Hcini et al., 2024; Rudroff et al., 2024; Salvi et al., 2025; Ursin et al., 2021). Concerns about accuracy, such as hallucination or prediction uncertainty, or potential use of fabricated data further increased the risks to patient safety and diagnostic reliability (El-Sappagh et al., 2023; Ford et al., 2023; Harrison et al., 2025; Hine et al., 2022; Lai et al., 2026; Rohrer et al., 2025; Ursin et al., 2021). Variability in system reliability (El-Sappagh et al., 2023; Lai et al., 2026; Rohrer et al., 2025; Salvi et al., 2025) and limited robustness of AI-based algorithms (El-Sappagh et al., 2023; Rudroff et al., 2024; Salvi et al., 2025) also raised concerns about robustness.

##### Respect for human dignity

The third theme was identified by ten reviews, which stated dignity-centered ethical considerations beyond technical and structural issues. Autonomy was the main issue discussed (Bhuiyan et al., 2025; de Aranha Martins, 2021; El-Sappagh et al., 2023; Harrison et al., 2025; Hernández-Torres & Sánchez-DelaCruz, 2025; Hine et al., 2022; Sætra, 2022; Ursin et al., 2021), followed by challenges in establishing or improving trust among patients, caregivers, and AI systems (Bhuiyan et al., 2025; de Aranha Martins, 2021; Graham et al., 2020; Hernández-Torres & Sánchez-DelaCruz, 2025) and potential psychosocioeconomic well-being, including psychological distress, family burden, financial strain, and social well-being (de Aranha Martins, 2021; El-Sappagh et al., 2023; Hine et al., 2022; Ursin et al., 2021). Moral decision-making capacity and the right to know about the diagnostic results (Ford et al., 2023; Ursin et al., 2021) and impersonal care, such as deception, authenticity, infantilization (de Aranha Martins, 2021; Hine et al., 2022; Sætra, 2022), were also reported. Reliance on AI with limited critical deployment (El-Sappagh et al., 2023) could further weaken relational care and undermine human dignity.

#### Environment and ecosystem flourishing

This domain states that AI development and deployment should avoid harming ecological systems and recognize environmental stability as a key component of human health and societal well-being (UNESCO, 2022). Considerably, the perspective of environmental well-being (e.g., environmental harm due to AI use, sustainability of AI, and environmental friendliness) has been addressed in only two included reviews (de Aranha Martins, 2021; El-Sappagh et al., 2023), which highlights that ethical discussions in dementia-related generative AI research tend to focus more on individual- and technology-level concerns than on environmental issues.

#### Ensuring diversity and inclusiveness

This domain focuses on fairness and equal participation (UNESCO, 2022). AI systems should mitigate exclusion, discrimination, and marginalization by promoting diversity in data, representation, and access, while respecting individuals’ different lifestyles, beliefs, and experiences regarding AI systems (UNESCO, 2022). This domain was reported in the theme of ‘equity and access.’

##### Equity and access

Ethical concerns related to health inequity and limited access were reported in sixteen reviews. A major concern was the lack of diversity in datasets, leading to low representativeness across populations (Bhuiyan et al., 2025; El-Sappagh et al., 2023; Hcini et al., 2024; Kale et al., 2024; Lai et al., 2026; Rudroff et al., 2024; Salvi et al., 2025). These less diverse datasets also caused algorithmic bias related to race, gender, cultural, and linguistic differences and further triggered risks of unequal outcomes (Bhuiyan et al., 2025; de Aranha Martins, 2021; El-Sappagh et al., 2023; Ford et al., 2023; Graham et al., 2020; Harrison et al., 2025; Hassan et al., 2025; Hernández-Torres & Sánchez-DelaCruz, 2025; Jiao, 2025; Ursin et al., 2021). A lack of fairness (El-Sappagh et al., 2023; Harrison et al., 2025; Hassan et al., 2025; Hcini et al., 2024; Hine et al., 2022; Rudroff et al., 2024), barriers to digital health access (Bhuiyan et al., 2025; Ford et al., 2023; Hassan et al., 2025; Hine et al., 2022), and structural health inequities (Ford et al., 2023; Hassan et al., 2025; Kale et al., 2024), particularly among economically and geographically underserved populations, exacerbated disparities in using AI systems in dementia care. Cost inequality and resource constraints further limited equitable implementation across dementia care environments (Salvi et al., 2025; Ursin et al., 2021).

#### Living in a peaceful, just, and interconnected society

The fourth domain discusses that AI systems should strengthen social cohesion, institutional trust, and solidarity while avoiding practices that cause inequality, social division, or power imbalances (UNESCO, 2021). This domain was associated with the theme ‘Governance.’

##### Governance

Fifteen included reviews revealed system validations, regulatory oversight, accountability mechanisms, and intellectual property. Regulatory oversight, including clear regulatory frameworks, tool validation, interoperability standards, risk stratification, clarified roles between humans and AIs, and common AI-related protocols, were frequently mentioned, indicating that governance and standardization are considered fundamental requirements for AI implementation (Bhuiyan et al., 2025; de Aranha Martins, 2021; El-Sappagh et al., 2023; Harrison et al., 2025; Hassan et al., 2025; Hernández-Torres & Sánchez-DelaCruz, 2025; Jiao, 2025; Kale et al., 2024; Lai et al., 2026; Rudroff et al., 2024; Salvi et al., 2025; Ursin et al., 2021). Additionally, accountability and liability complexity reflected potential ethical issues associated with AI integration (de Aranha Martins, 2021; El-Sappagh et al., 2023; Ford et al., 2023; Graham et al., 2020; Harrison et al., 2025; Rohrer et al., 2025; Salvi et al., 2025; Ursin et al., 2021). Intellectual property rights added further complexity to data governance (Kale et al., 2024). These components align with the institutional and social environment for broader responsible AI use and are essential for sustaining public trust and developing a regulatory framework for dementia AI deployment.

### Mapping identified categories to bioethical principles

Along with the UNESCO AI values, the identified ethical categories were mapped to the traditional bioethical principles of respect for autonomy, beneficence, non-maleficence, and justice (Table 3). Concerns related to privacy and informed consent within the theme of protections of human rights, transparency and explainability within technical trustworthiness, and autonomy and moral decision-making within the respect for human dignity were aligned with the principle of respect for autonomy.

However, several categories also overlapped with other bioethical principles. Categories associated with the protection of human rights emphasized the prevention of harm (non-maleficence), whereas categories under technical trustworthiness were associated with beneficence due to their focus on improving healthcare quality, safety, and reliability. Within the theme of respect for human dignity, trust and moral decision-making were more closely associated with beneficence, while psychosocioeconomic well-being, impersonal care, and reliance on AI without limited critical deployment were more closely aligned with non-maleficence.

Categories under the theme of equity and access were closely aligned with the bioethical principle of justice, which emphasizes the fair distribution of healthcare resources and benefits in healthcare systems, particularly for vulnerable populations. Accountability within the theme of governance was also related to justice, while regulatory oversight was more closely associated with non-maleficence. Intellectual property within the governance theme, as well as environmental well-being, were less readily classified within the four traditional bioethical principles. This suggests the need for a broader ethical principle that better captures technical as well as social dimensions of AI in healthcare.

### Quality appraisal

The quality appraisal scores varied across the included reviews (see Table 4), ranging from two to nine out of 11. Studies included in this review of reviews clearly explained the review purpose and questions and provided recommendations and implications for policy, practice, and new research approaches based on their findings. Only about half of the reviews addressed core methodological components, including appropriate inclusion criteria and search strategies, data sources for identifying studies related to their topic, and suitable methods for synthesizing findings. Only five reviews reported using appropriate criteria for study appraisal or described procedures to minimize data extraction errors, indicating limited attention to quality control processes. Furthermore, independent appraisal by multiple reviewers and assessment of publication bias were not reported except for one review (Rohrer et al., 2025), raising concerns about the overall reliability and transparency of the evidence base.

**Table 4:** Quality appraisal using JBI tool (Aromataris et al., 2015).

Author (year)	Is the review question clearly and explicitly stated?	Were the inclusion criteria appropriate for the review question?	Was the search strategy appropria-te?	Were the sources and resources used to search for studies adequate?	Were the criteria for appraising studies approp-riate?	Was critical appraisal conducted by two or more reviewers independe-ntly?	Were there methods to minimize errors in data extraction?	Were the methods used to combine studies appropriate?	Was the likelihood of publicati-on bias assessed?	Were recommenda-tions for policy and/or practice supported by the reported data?	Were the specific directives for new research appropri-ate?
Bhuiyan et al. (2025)	Yes	No	No	No	No	No	No	N/A	No	Yes	Yes
de Aranha Martins (2021)	Yes	Yes	Yes	No	No	No	No	Yes	No	Yes	Yes
El-Sappagh et al. (2023)	Yes	Yes	Yes	Yes	Yes	No	Yes	Yes	No	Yes	Yes
Ford et al. (2023)	Yes	No	No	No	No	No	No	Yes	No	Yes	Yes
Graham et al. (2020)	Yes	Unclear	Unclear	Unclear	N/A	No	N/A	N/A	No	Yes	Yes
Harrison et al. (2025)	Yes	No	No	No	No	No	No	N/A	No	Yes	Yes
Hassan et al. (2025)	Yes	Yes	Yes	Yes	Yes	No	No	Yes	No	Yes	Yes
Hcini et al. (2024)	Yes	Yes	Yes	Yes	No	No	No	Yes	No	Yes	Yes
Hernández-Torres and Sánchez-DelaCruz (2025)	Yes	Yes	Yes	Yes	Yes	No	No	Yes	No	Yes	Yes
Hine et al. (2022)	Yes	N/A	N/A	N/A	N/A	N/A	N/A	N/A	N/A	Yes	Yes
Jiao (2025)	Yes	Yes	Yes	Yes	Yes	No	No	Yes	No	Yes	Yes
Kale et al. (2024)	Yes	No	Unclear	No	No	No	No	N/A	No	Yes	Yes
Lai et al. (2026)	Yes	Yes	Yes	Yes	No	No	Yes	Yes	No	Yes	Yes
Rohrer et al. (2025)	Yes	Yes	Yes	Yes	No	Yes	Yes	Yes	N/A	Yes	Yes
Rudroff et al. (2024)	Yes	No	Unclear	Unclear	No	No	No	N/A	No	Yes	Yes
Sætra (2022)	Yes	N/A	N/A	N/A	N/A	N/A	N/A	N/A	N/A	Yes	N/A
Salvi et al. (2025)	Yes	Yes	Yes	Yes	Yes	Unclear	Yes	Yes	No	Yes	Yes
Ursin et al. (2021)	Yes	Yes	Yes	Yes	No	No	Yes	Yes	No	Yes	Yes

*Note.* JBI = Joanna Briggs Institute; N/A = not applicable.

Risk of bias varied across the included reviews (see Table 5). Reviews with JBI scores below 6 generally showed more methodological concerns when it comes to ratings in eligibility criteria and study identification, synthesis methods, and how conclusions were supported by the evidence. In contrast, reviews with JBI scores of 6 or above tended to show lower risk of bias in these parts, although several still had high-risk ratings for data collection and risk-of-bias assessment procedures. Overall, these findings suggest that while higher-quality reviews were generally more methodologically robust, concerns remained in some domains, particularly around how data were collected, extracted, and appraised.

**Table 5:** Risk of bias using ROBIS tool (Whiting et al., 2016).

Quality of JBI	Author (year)	Describe the study eligibility criteria, any restrictions on eligibility and whether there was evidence that objectives and eligibility criteria were pre-specified	Describe methods of study identification and selection	Describe methods of data collection, what data were extracted from studies or collected through other means, how risk of bias was assessed and the tool used to assess risk of bias	Describe synthesis methods	Describe whether conclusions were supported by the evidence
Below 6	Bhuiyan et al. (2025)	UC	UC	H	UC	UC
	Ford et al. (2023)	H	H	H	H	H
	Graham et al. (2020)	H	UC	H	H	H
	Harrison et al. (2025)	UC	UC	H	UC	H
	Hine et al. (2022)	UC	UC	UC	UC	H
	Kale et al. (2024)	UC	UC	H	UC	H
	Rudroff et al. (2024)	UC	UC	H	UC	H
	Sætra (2022)	UC	UC	H	UC	UC
6 or above	de Aranha Martins (2021)	L	UC	H	UC	H
	El-Sappagh et al., (2023)	L	L	L	L	L
	Hassan et al. (2025)	L	L	UC	H	L
	Hcini et al. (2024)	L	L	H	L	L
	Hernández-Torres and Sánchez-DelaCruz (2025)	L	L	L	UC	H
	Jiao (2025)	L	L	H	L	L
	Lai et al. (2026)	L	L	H	L	L
	Rohrer et al. (2025)	L	L	H	L	L
	Salvi et al. (2025)	L	L	H	L	L
	Ursin et al. (2021)	L	L	L	L	L

*Note.* H = high risk; L = low risk; UC = unclear; ROBIS = the Risk of Bias in Systematic Reviews Tool; JBI = Joanna Briggs Institute.

## Discussion

This review of reviews identified ethical challenges highlighted in previous reviews on AI systems used in dementia care. According to UNESCO’s four domains of AI value (UNESCO, 2022), these ethical concerns were categorized into five themes: protection of human rights (e.g., privacy, security, malicious use, and informed consent), technical trustworthiness (e.g., transparency, explainability, accuracy, reliability, and robustness), respect for human dignity (e.g., autonomy, trust, moral decision making, psychosocioeconomic harm, impersonal care, and overreliance), environmental well-being, equity and access (e.g., dataset diversity, algorithmic bias, fairness, structural inequalities, digital divide, costs and resources), and governance (e.g., regulatory oversight, accountability, and intellectual property). Most ethical issues centered on protecting human rights and dignity, followed by diversity, inclusiveness, and fostering a peaceful, interconnected society. Environmental and ecosystem concerns were mostly overlooked.

Ethical concerns in AI-supported dementia care encompassed not only human and technical issues specific to AI but also wider concerns, such as equity, accountability, and the well-being of humans, society, and the environment. These concerns indicate that while AI has the potential to improve dementia diagnosis, management, and caregiving, its use may also expand the scope of ethical considerations, including healthcare disparities, inadequate data governance, and weakened relational aspects of care. A socio-technical trustworthiness framework explains how those involved in developing and deploying technologies are interconnected in influencing technology design, and that this interconnection also mutually affects environmental factors, including sociocultural and legislative conditions (Paramonova et al., 2024). This perspective highlights that ethical considerations across multiple areas are essential for advancing AI overall and are often overlooked when the focus is focused on technical performance only.

Caregiving-related concerns indicate that AI should not replace relational, human-centered care. Dementia care is typically relational and dyadic, and caregivers carry various responsibilities for care recipients and maintain stability while navigating role dynamics, challenges, and decision-making (Kårelind et al., 2026; Köhler et al., 2022). Reported ethical considerations in this review of reviews, such as overreliance, psychological impact, and impersonal care, suggest that AI can influence caregiving roles, responsibilities, and dyadic relationships. Overlooking caregiving aspects in AI development and deployment would narrow the analysis of autonomy and dignity to patients alone, without considering relational contexts. Therefore, AI use should be considered a complementary approach to supporting clinicians and caregivers while preserving human dignity and relational care.

Although concerns were reported across multiple categories, the included reviews focused less on how AI use could influence human and social values across various populations. From a UNESCO perspective, these concerns were closely related to human dignity (UNESCO, 2022); still, there remains limited discussion on how these AI systems can be meaningfully extended across diverse populations and social contexts. Limited attention to diverse populations, including underserved populations (e.g., those with low income, older adults, and people living in rural areas), would cause limited, biased datasets that may hinder the effectiveness of the AI-based tools for care, reducing applicability (Bhuiyan et al., 2025; Harrison et al., 2025; Rudroff et al., 2024; Salvi et al., 2025). To consider this gap, future research should involve patients, caregivers, and marginalized communities and integrate the potential impact of AI use into development pipelines (Salvi et al., 2025). It may also be important to use diverse and balanced training data, improve the model by reducing biases and filtering biased outputs during and after training, and guiding the model to check and correct itself (Harrison et al., 2025).

Furthermore, issues related to environmental and ecosystem health were largely overlooked in the reviews included on AI in dementia care. The lack of focus on environmental factors suggests that researchers have prioritized immediate patient rights and clinical effectiveness over long-term sustainability. AI in healthcare can lead to high energy use, greenhouse gas emissions, and electronic waste (Ueda et al., 2024). Dementia care technologies often focus on continuous monitoring systems, cloud-based analytics, and sensor-based observations (Salvi et al., 2025), which contribute to environmental problems. Given the long-term nature of dementia care, overlooking the environmental impacts of AI might increase ecological costs for future generations. Therefore, the ecological footprint and long-term demands of AI systems should be considered in the ethical evaluation of dementia research. Addressing this gap could help researchers improve innovation in dementia care and expand commitments to sustainable, responsible AI development.

Our findings partially align with a review on healthcare AI suggesting that the four principles of biomedical ethics are broadly applicable to many AI-related concerns (Gorelik et al., 2025). Similarly, our analysis also found that many categories based on AI use could be interpreted through these traditional bioethical principles. However, our findings reported the limited applicability of traditional bioethical principles to some specific issues. For example, categories such as intellectual property and environmental well-being, which were less likely to address human-centered considerations, did not fall under the bioethical principles. This difference may reflect our use of the UNESCO AI values as an additional analytic framework, which includes broader concerns beyond the human-centered scope of the four bioethical principles. This can also be supported by previous researchers arguing that a principle-based ethics framework alone may be insufficient for guiding AI implementation in healthcare (Benzinger et al., 2023; Heuser et al., 2025). Therefore, future ethical frameworks for AI in dementia care should add technology ethics, governance principles, and environmental perspectives to human-centered bioethics to better address the complexity of AI implementation in healthcare.

### Implications for clinical practice

The findings highlighted important implications for clinical practice in dementia care. Nurses often serve as mediators between technology, people living with dementia, and their family caregivers, making them closely involved in the ethical tensions raised by AI use. This includes interpreting AI-informed outputs, communicating uncertainty, protecting privacy, supporting consent processes, and balancing safety with autonomy and dignity (Harishbhai Tilala et al., 2024). These responsibilities are especially vital in dementia care, where cognitive vulnerability, decision-making complexity, and the relational context of care create uniquely profound ethical demands on AI system. The included reviews suggest that concerns about autonomy, informed consent, transparency, trust, and potential harm are particularly critical in dementia, because AI-informed outputs can influence how people living with dementia are assessed, monitored, and understood in ways that are not value-neutral (Bhuiyan et al., 2025; Ford et al., 2023; Harrison et al., 2025; Kale et al., 2024; Rudroff et al., 2024; Ursin et al., 2021). Therefore, care should not be reduced to algorithmic prediction or system efficiency alone. AI may support assessment, monitoring, and care planning, but its use remains ethically acceptable only when it enhances, rather than weakens, dignity, autonomy, privacy, and relational care within person-centered fundamental care (de Aranha Martins, 2021; Hine et al., 2022; Sætra, 2022; Ursin et al., 2021).

### Implications for research and policy

In research, the findings emphasize the importance of developing dynamic models that include users, developers, and AI systems to enhance transparency, explainability, and accuracy of AI. They also need to address health equity in AI use, not just regarding algorithmic bias but also accessibility, affordability, and real-world inclusion. At the policy level, governance-related concerns underscore the need for clear regulatory frameworks, standardized validation processes, and well-defined accountability mechanisms, along with attention to environmental considerations, to ensure the safe and sustainable deployment of AI.

### Strengths

This study reviews and synthesizes existing reviews on AI ethics in dementia and caregiving research. By revisiting prior literature, our review can identify shared conceptual consistencies and research gaps across the literature. Rather than focusing on technical performance, this review explores ethical questions about how AI-based tools are used in dementia care and highlights how ethical considerations often receive less attention than effectiveness. This perspective helps researchers and healthcare professionals better recognize ethical risks that require a careful, proactive approach throughout the design, development, and implementation of AI systems. Additionally, by comparing how ethical concerns vary across different contexts, this study can promote more ethically responsive innovation and guide the development of governance strategies, clinical protocols, and regulatory approaches tailored to dementia and caregiving environments.

### Limitations

Despite its strengths and implications, this review has several limitations. First, as a review of reviews, the analysis may not fully capture the specific contexts, data characteristics, or implementation conditions of individual primary studies, although synthesizing existing reviews allows for a broader conceptual overview. Second, ethical issues were not categorized by specific AI modalities or system types because the ethical concerns were generally discussed at a high level of abstraction. Future research should consider classifying ethical issues by context. Third, the overall methodological quality of the included reviews was relatively low. Variations in search strategies, inclusion criteria, and quality appraisal procedures among the included reviews may affect the robustness of the synthesized findings in this review of reviews.

## Conclusion

This review of reviews synthesized ethical considerations related to AI in dementia care using UNESCO’s AI ethics value domains. The identified key ethical considerations may influence people living with dementia and their caregivers and underscore the need for more responsible development and implementation of AI technologies in dementia care. The findings of this review may further provide a foundation for embedding ethical principles more carefully within multifaceted approaches and for guiding research to develop AI tools that support dementia care in safer, more ethically responsible ways.

## Supplementary Material

gnag158_Supplementary_Data

## Data Availability

As this is a review article, no new data were generated or analyzed, and therefore data sharing is not applicable to this article.

## Protocol registration

The protocol of this review is registered in Open Science Framework (https://osf.io/24fd9).
