# Supplementary material for: Ethical considerations of artificial intelligence in dementia research: a review of reviews
Source: Gerontologist. 2026 Jul 23;66(10):gnag158. doi: 10.1093/geront/gnag158 (PMC13592678; doi:10.1093/geront/gnag158)
Supplement: Supplementary file 1 [file gnag158_supplementary_data.zip › Supp_Appendix A.docx]

**Supplement Appendix A**

PUBMED

| **#** | **Query** | **# of Reviews** |
| --- | --- | --- |
| 1 | "cognitive dysfunction"[MeSH Terms] OR "cognitive dysfunction"[MeSH Terms] OR "cognitive dysfunction"[MeSH Terms] OR "dementia"[MeSH Terms] OR "alzheimer disease"[MeSH Terms] OR "dementia"[Text Word] OR "alzheimer*"[Text Word] OR "alzheimer s disease"[Text Word] | 493,918 |
| 2 | (caregiver[MeSH Terms]) OR (carer[MeSH Terms]) OR (caregiver*[Text Word]) OR (carer*[Text Word]) OR (care giver[Text Word]) | 150,726 |
| 3 | ((ethics [Mesh] OR "ethic*" OR "codes of ethics" OR "autonomy" OR "benevolence" OR "non-maleficence" OR "nonmaleficence" OR "justice") OR (ethic[Text Word]) OR (autonomy[Text Word]) OR (benevolence[Text Word]) OR (non-maleficence[Text Word]) OR (justice[Text Word]))) | 686,336 |
| 4 | (("Machine Learning"[Text Word] OR "Natural Language Processing"[Text Word] OR "Artificial Intelligence"[Text Word] OR "Natural Language Processing"[MeSH Terms] OR "Machine Learning"[MeSH Terms] OR "Artificial Intelligence"[MeSH Terms] OR "AI" OR "Deep learning")) | 910,666 |
| 5 | 1 or 2 | 530,960 |
| 6 | 3 and 4 and 5 | 806 |

EMBASE

| **#** | **Query** | **# of Reviews** |
| --- | --- | --- |
| 1 | exp cognitive defect/ or exp dementia/ or exp alzheimer's disease/ or (cognitive defect or dementia or alzheimer's disease or alzheimer* or cognitive decline or cognitive impairment).mp. | 847,960 |
| 2 | exp caregiver/ or (carer* or caregiver* or care giver*).mp. | 233,256 |
| 3 | exp ethics/ or (ethic* or autonomy or benevolence or non-maleficence or nonmaleficence or justice).mp. | 690,916 |
| 4 | exp machine learning/ or exp artificial intelligence/ or exp deep learning/ or exp deep learning/ or (machine learning or natural language processing or artificial intelligence or AI or machine learning or deep learning).mp. | 841,560 |
| 5 | 1 or 2 | 1,041,921 |
| 6 | 3 and 4 and 5 | 716 |

PsycINFO

| **#** | **Query** | **# of Reviews** |
| --- | --- | --- |
| 1 | exp cognitive impairment/ or exp dementia/ or exp alzheimer's disease/ or (dementia or alzheimer's disease or alzheimer* or cognitive decline or cognitive impairment).mp. | 185,915 |
| 2 | exp caregivers/ or (carer* or caregiver* or care giver*).mp. | 95,254 |
| 3 | exp Robot Ethics/ or exp Artificial Intelligence Ethics/ or exp Ethics/ or (ethic* or autonomy or benevolence or non-maleficence or nonmaleficence or justice).mp. | 228,871 |
| 4 | exp machine learning/ or exp artificial intelligence/ or exp deep neural networks/ or exp natural language processing/ or (machine learning or natural language processing or artificial intelligence or AI or machine learning or deep learning).mp. | 98,429 |
| 5 | 1 or 2 | 263,889 |
| 6 | 3 and 4 and 5 | 188 |

ProQuest

| **#** | **Query** | **# of Reviews** |
| --- | --- | --- |
| 1 | summary(cognitive impairment) OR summary(cognitive decline) OR summary(dementia) OR summary(cognitive defect) OR summary(alzheimer*) OR summary(alzheimer's disease) | 691,207 |
| 2 | summary(caregiver*) OR summary(carer*) OR summary(care giver*) | 443,522 |
| 3 | summary(ethic*) OR summary(autonomy) OR summary(benevolence) OR summary(non-maleficence) OR summary(nonmaleficence) OR summary(justice) | 5,300,045 |
| 4 | summary(machine learning) OR summary(artificial intelligence) OR summary(AI) OR summary(natural language processing) OR summary(deep learning) | 1,502,571 |
| 5 | 1 or 2 | 1,085,491 |
| 6 | 3 and 4 and 5 | 432 |

CINAHL

| **#** | **Query** | **# of Reviews** |
| --- | --- | --- |
| 1 | ( MH "Cognitive Decline" OR MH "Cognitive Impairment" OR MH "Cognition Disorders" OR MH "Dementia" OR MH "Alzheimer Disease" OR TX dement* OR TX alzheimer* OR TX "cognitive decline" OR TX "cognitive impairment" OR TX "cognitive dysfunction" ) | 176,115 |
| 2 | (MH "Caregivers" OR TX caregiver* OR TX carer* OR TX "care giver") | 118,862 |
| 3 | (MH "Ethics" OR MH "Ethical Principles" OR MH "Autonomy" OR MH "Beneficence" OR MH "Justice" OR TX ethic* OR TX autonomy OR TX benevolence OR TX "non-maleficence" OR TX nonmaleficence OR TX justice) | 225,343 |
| 4 | (MH "Artificial Intelligence" OR MH "Machine Learning" OR MH "Natural Language Processing" OR TX "artificial intelligence" OR TX "machine learning" OR TX "natural language processing" OR TX "AI" OR TX "deep learning") | 85,358 |
| 5 | 1 or 2 | 274,866 |
| 6 | 3 and 4 and 5 | 141 |

IEEE

| **#** | **Query** | **# of Reviews** |
| --- | --- | --- |
| 1 | "cognitive decline" OR "cognitive impairment" OR dement* OR "Alzheimer's disease" OR alzheimer* OR dementia  OR caregiver* OR carer* OR "family caregiver" | 15,537 |
| 2 | ethic* OR autonomy OR "responsible AI" OR "data governance" | 42,496 |
| 3 | "artificial intelligence" OR "machine learning" OR "deep learning"  OR "natural language processing" OR NLP OR AI | 790,021 |
| 4 | 1 and 2 and 3 | 190 |
